# Informed Choice in the German Mammography Screening Program by Education and Migrant Status: Survey among First-Time Invitees

**DOI:** 10.1371/journal.pone.0142316

**Published:** 2015-11-03

**Authors:** Eva-Maria Berens, Maren Reder, Oliver Razum, Petra Kolip, Jacob Spallek

**Affiliations:** 1 Department of Epidemiology & International Public Health, School of Public Health, Bielefeld University, Bielefeld, North-Rhine Westphalia, Germany; 2 Department of Health Services Research and Nursing Science, School of Public Health, Bielefeld University, Bielefeld, North-Rhine Westphalia, Germany; 3 Department of Prevention and Health Promotion, School of Public Health, Bielefeld University, Bielefeld, North-Rhine Westphalia, Germany; 4 Department of Public Health, Brandenburg University of Technology Cottbus-Senftenberg, Brandenburg, Germany; The University of Hong Kong, HONG KONG

## Abstract

Breast cancer is the most prevalent cancer among women and mammography screening programs are seen as a key strategy to reduce breast cancer mortality. In Germany, women are invited to the population-based mammography screening program between ages 50 to 69. It is still discussed whether the benefits of mammography screening outweigh its harms. Therefore, the concept of informed choice comprising knowledge, attitude and intention has gained importance. The objective of this observational study was to assess the proportion of informed choices among women invited to the German mammography screening program for the first time. A representative sample of 17,349 women aged 50 years from a sub-region of North Rhine Westphalia was invited to participate in a postal survey. Turkish immigrant women were oversampled. The effects of education level and migration status on informed choice and its components were assessed. 5,847 (33.7%) women responded to the postal questionnaire of which 4,113 were used for analyses. 31.5% of the women had sufficient knowledge. The proportion of sufficient knowledge was lower among immigrants and among women with low education levels. The proportion of women making informed choices was low (27.1%), with similar associations with education level and migration status. Women of low (OR 2.75; 95% CI 2.18–3.46) and medium education level (OR 1.49; 95% CI 1.27–1.75) were more likely to make an uninformed choice than women of high education level. Turkish immigrant women had the greatest odds for making an uninformed choice (OR 5.30, 95% CI 1.92–14.66) compared to non-immigrant women. Other immigrant women only had slightly greater odds for making an uninformed choice than non-immigrant women. As immigrant populations and women with low education level have been shown to have poor knowledge, they need special attention in measures to increase knowledge and thus informed choices.

## Introduction

Population-based breast cancer screening programs have been implemented in many European countries in the last decades. In Germany, women aged 50 to 69 years are invited for a mammogram in a specialized center every two years [1]. The aims of such programs are to reduce mortality from breast cancer and improve outcomes through early detection and treatment [2]. However, mammography screening also has negative implications such as false positive results, overdiagnosis and overtreatment (e.g. [3]). As mammography screening addresses healthy women, informed choice is crucial. According to the European guidelines for quality assurance in mammography screening, every woman should know the benefits and risks of screening and make her own decision for or against participation [2]. An “informed choice” is based on (1) knowledge, (2) personal attitudes, and (3) intention and actual behavior [4]. Although the concept of informed choice competes with the goal of a high participation rate (at least 70 per cent to assure quality and effectiveness of the screening) [2], its fundamental importance is now accepted by most stakeholders in Germany [5].

Previous studies from various European countries on participation in mammography screening have mostly focused on general determining factors of screening participation aiming to increase participation. Age, income, occupation, education level, number of children, housing property, place of residence and geographic distance to the screening unit have been shown to influence mammography screening participation (e.g. [6–8]). Immigrant women are less likely to participate in mammography screening than women of the respective autochthonous population in several European countries [9–15], but not in Germany [16]. Knowledge about mammography screening is low, with only 1.5 percent of all women in Europe correctly knowing the estimated reduction in breast cancer mortality achieved through regular attendance of screening [17]. In a study among women aged 44 to 63 years in Germany, benefits of mammography screening tended to be overestimated and risks underestimated [18]. In the Netherlands, the proportion of informed choices is higher with 88 per cent of first-time-invitees making an informed choice about mammography screening [19]. In Germany, there are no studies considering informed choice among women invited to the mammography screening program for the first time. Even data on the components of informed choice among immigrant women and by education level is scarce.

The aim of the study is to assess the proportion of women making an informed choice in the German population-based mammography screening program. A special focus is on possible differences in informed choice among women of different education level and migration status.

## Materials and Methods

The InEMa (In*formierte* E*ntscheidung zur Teilnahme am* Ma*mmographie-Screening-Programm*) study is an observational study investigating informed choice in women invited to the German nation-wide population-based mammography screening program for the first time.

### Study population and design

Women aged 50 years living in Westphalia-Lippe, a sub-region of the Federal State of North-Rhine Westphalia in Germany, were invited to participate in the study. A postal questionnaire (see S1 File: German questionnaire and S2 File: Turkish questionnaire) was sent to the women one to two months after their 50^th^ birthday. At this time women receive their invitation with a pre-specified appointment (date and time) to the mammography screening program through the regional mammography organization, though a large variability exists in invitation timing. Data were collected between October 2013 and July 2014.

A random sample was drawn from registration offices, comprising 15,561 non-Turkish immigrant women (equivalent to 54 per cent of such women in the study area). Turkish immigrants were identified by a name-based approach [20], defining all women as Turkish who have a Turkish name, independent of their migration history or nationality. All 1,789 women who were identified as likely to be Turkish were invited to participate to allow oversampling. They received all materials in German and in Turkish. A bilingual reminder postcard was sent to all women one week after the study invitation. The study has two data collection points (see the study protocol for further details [21]). We here present data from the first data collection point preceding a possible screening appointment.

Women who ever had a breast cancer diagnosis were excluded from the analyses as they are not part of the target population for screening. Women who already participated in the screening program were also excluded as no intention to attend at the first contact could be assessed. Furthermore, women who had not received an invitation for the national mammography screening program at the time of the study were excluded as they had not been exposed to the official information materials.

The study was approved by the ethical committee of the Medical Faculty of Muenster University (2012-268-f-S) and the Data Protection Officer of Bielefeld University. Individual data (names and addresses) were stored separately from the questionnaire data. Women gave written consent to participate in the study.

### Outcome variables

The primary outcome of the study was informed choice, which comprises three dimensions: knowledge (sufficient/insufficient), attitude (positive/negative), and intention (yes/no).

The questionnaire we developed is based on existing instruments, qualitative Interviews with German and Turkish women and a qualitative study on factors related to mammography screening participation among Turkish women [22]. It included many different components (see the study protocol [21]). The relevant components for this publication are described below.

#### Informed choice

Informed choice was defined as stating an intention regarding (non-)participation in mammography screening which is (a) based on sufficient knowledge and (b) congruent with one’s attitude. This second criterion, in turn, was defined as (b1) having a positive attitude and intending to participate in the national mammography screening program or (b2) having a negative attitude and neither intending to participate in the program nor in opportunistic screening. All other combinations of knowledge, attitude and intention were classified as uninformed. The index of informed choice was thus calculated from the attitude scale, the knowledge index and the items on intention (all are separately described below). Our results suggest that the attitude scale and the knowledge index have favorable psychometric properties.

#### Attitude

The attitude towards mammography screening was measured based on the Reasoned Action Approach [23]. The scale consisted of four items. These semantic differentials, which have been used by Marteau [4] in the context of antenatal screening, were applied to the context of mammography screening. Women were asked to rate the statement ‘To participate in the MSP is…’ on four semantic differentials (5-point scale ranging from -2 to +2): ‘Important/not important’, ‘a good thing/a bad thing’, ‘comfortable/uncomfortable’, ‘beneficial/harmful’. A score of equal to or larger than zero (possible score range -8 to +8) was classified as positive attitude.

#### Knowledge

The knowledge index comprised seven multiple choice questions based on knowledge questionnaires of Mathieu [24] and Marteau [4]. The following topics were included: target population of mammography screening, frequency and meaning of a positive screening result, incidence and mortality with or without screening program, false-negative results, and overtreatment. A score of 1 indicated a correct answer, a score of 0 an incorrect answer. Missing responses and “Don’t know” responses were categorized as incorrect. There are no agreed criteria for defining sufficient knowledge, so we used the midpoint of the scale as cut-off point. This has been applied previously [19]. Thus, a score of larger than 3 (possible score range 0 to 7) was categorized as having sufficient knowledge.

#### Mammography intention

Intention to participate in mammography screening was assessed by two newly developed questions. This was necessary to reflect the German context in which an organized screening program with automatic invitation works in parallel to opportunistic screening. Firstly, women were asked about their general intention to receive mammography screening in the next three months. Three months was chosen as a reasonable time frame because the women already had received the invitation with the appointment. Secondly, women intending to receive mammography were asked whether they planned to undergo screening in the organized screening program or elsewhere (so called opportunistic screening by gynecologists or radiologists). Women intending to receive a screening mammography but not responding to the second question were categorized as ‘yes, not specified’. For the categorization of informed choice women with intention to perform opportunistic screening or unspecified intention were excluded.

#### Predictor variables

Education level and migration. Migration status and education level were included as independent variables in the analyses. Migration status was defined by country of birth as immigrant women aged 50 are unlikely to be second generation immigrants.

The group of ‘resettlers’ are special immigrants in Germany as they are ethnic Germans who emigrated to Russia and other Eastern European countries in the 17^th^-19^th^ century; most return-migrated to Germany after the collapse of the Soviet Union. Resettlers ‘automatically’ get German nationality. Women who were born in an Eastern European country and had a self-reported resettler-status were categorized as ‘resettlers’. Results are shown for non-immigrant women (autochthonous population) and the immigrant groups of resettlers, Turkish women and women born in other countries.

Education level was assessed by years of school education or degree. Women without a degree or a degree equivalent to a maximum of nine years at school are categorized as low education level *(Haupt-/Volksschule)*. Women with ten years of education (*Realschule/Polytechnische Oberschule*) were categorized as medium level, and women with at least 12 years of education *(Abitur/Fachabitur)* were categorized as high education level which is comparable to A-levels.

### Statistical analysis

Data were analyzed using SPSS 22.0. The proportion of missing values was very low (<2%) except for ‘intention to participate’. Therefore, we did not perform imputation procedures as originally planned in the study protocol. For intention, we chose to include a missing response as a separate category.

Descriptive analyses to characterize the study population were performed stratified by education level and migration status (Table 1). The distribution-matrix of the different outcomes of the components of informed choice (knowledge, attitude, intention) was not stratified by education or migration due to small numbers in some cells (Table 2). The percentage of informed choices was calculated only (1) for women with no intention to perform mammography in the next three months, and (2) for women intending to receive a screening mammography within the population-based screening program (Tables 3 and 4). Women intending to undergo opportunistic screening were excluded because knowledge and attitude questions focused on the screening program. The proportion of women who had made informed choices (i.e. sufficient knowledge and consistency between attitude and intention) were described stratified by education level and migration status. Group differences were assessed by chi-square tests (Table 3). Finally, odds ratios and 95% confidence intervals were computed to examine the effects of education level and migration on informed choice using univariate and adjusted logistic regression (Table 4). Due to small numbers, interaction between migration and education level could not be assessed.

**Table 1:** Intention to take up mammography screening, attitude, and knowledge, by level of education and migration status.

	% (n)	Intentional mammography screening uptake % (n)					Attitude	Knowledge
		No % (n)	Yes, opportunistic % (n)	Yes, screening program % (n)	Yes, not specified % (n)	Missing % (n)	(-8 to +8) Mean (SD)	(0 to 7) Mean (SD)
Education level*								
*High*	*36*.*3 (1*,*492)*	*15*.*5 (232)*	*5*.*5 (82)*	*73*.*3 (1*,*094)*	*3*.*3 (49)*	*2*.*3 (35)*	*3*.*88 (2*.*83)*	*3*.*14 (1*.*42)*
*Medium*	*41*.*4 (1*,*704)*	*13*.*4 (228)*	*5*.*1 (87)*	*74*.*8 (1*,*274)*	*4*.*3 (74)*	*2*.*4 (41)*	*4*.*29 (2*.*70)*	*2*.*84 (1*.*42)*
*Low*	*21*.*0 (863)*	*10*.*5 (91)*	*5*.*1 (44)*	*74*.*3 (641)*	*3*.*0 (26)*	*7*.*1 (61)*	*4*.*74 (2*.*48)*	*2*.*23 (1*.*32)*
Migration**								
*Non-immigrant*	*89*.*8 (3*,*695)*	*13*.*7 (507)*	*5*.*1 (190)*	*74*.*6 (2*,*755)*	*3*.*7 (136)*	*2*.*9 (107)*	*4*.*18 (2*.*74)*	*2*.*87 (1*.*43)*
*Resettlers*	*4*.*4 (179)*	*8*.*9 (16)*	*6*.*7 (12)*	*73*.*2 (131)*	*1*.*1 (2)*	*10*.*1 (18)*	*4*.*67 (2*.*42)*	*2*.*41 (1*.*33)*
*Turkish*	*2*.*5 (104)*	*16*.*3 (17)*	*8*.*7 (9)*	*60*.*6 (63)*	*5*.*8 (6)*	*8*.*7 (9)*	*5*.*07 (2*.*95)*	*1*.*73 (1*.*12)*
*Others*	*3*.*2 (133)*	*15*.*8 (21)*	*5*.*3 (7)*	*66*.*9 (89)*	*5*.*3 (7)*	*6*.*8 (9)*	*4*.*81 (2*.*30)*	*2*.*46 (1*.*49)*
Total	(4,113)	13.6 (561)	5.3 (218)	73.9 (3,040)	3.7 (151)	3.5 (143)	4.24 *(2*.*72)*	2.81 *(1*.*44)*

*54 Missings (1.3 percent);

** 2 Missings (<0.1 percent)

**Table 2:** Proportion of women by dichotomous dimensions of informed choice.

Knowledge	Attitude	Positive intention	
		No	Yes, screening program
Sufficient	Negative	1.5 *(54)* ^a^	0.9 *(33)* ^b^
	Positive	3.5 *(125)* ^b^	25.6 *(922)* ^a^
Insufficient	Negative	2.7 *(96)*	1.2 *(45)* ^b^
	Positive	7.9 *(286)* ^b^	56.7 *(2040)*

Note: data are given as percent (number); n = 3,601

^a^ defined as informed choice

^b^ inconsistency between attitude and intentional mammography uptake

**Table 3:** Proportion of sufficient knowledge, consistency, and informed choice, by education level and migration.*

	Sufficient knowledge		Consistency between attitude and intention		Informed choice	
Education level^a^						
*high*	39.9 *(529)*	p<0.001	86.2 *(1143)*	p = 0.41	34.6 *(459)*	p<0.001
*medium*	31.4 *(471)*		86.0 *(1292)*		26.5 *(398)*	
*low*	17.5 *(128)*		88.0 *(644)*		15.6 *(114)*	
Migration^b^						
*Non-immigrant*	32.7 *(1068)*	p<0.001	86.6 *(2826)*	p = 0.25	28.1 *(918)*	p<0.001
*Resettlers*	23.1 *(34)*		87.8 *(129)*		21.8 *(32)*	
*Turkish*	5.0 *(4)*		81.3 *(65)*		5.0 *(4)*	
*Others*	25.5 *(28)*		81.8 *(90)*		20.0 *(22)*	
Total^c^	31.5 *(1134)*		86.4 *(3112)*		27.1 *(976)*	

Note: data are given as percent (number), p-values from chi-square tests

*Informed choice for women with intention to participate in mammography screening program or no intention to perform mammography

^a^ n = 3,560

^b^ n = 3,599

^c^ n = 3,601

**Table 4:** Determinants of uninformed choice: results of logistic regression modelling.*

	OR	95% CI	p-value
*univariate*			
Education level^a^			
*high*	Ref.		
*medium*	1.47	1.25–1.73	<0.001
*low*	2.87	2.28–3.61	<0.001
Migration^b^			
*Non-immigrant*	Ref.		
*Resettlers*	1.41	0.95–2.10	0.093
*Turkish*	7.44	2.72–20.40	<0.001
*Others*	1.57	0.98–2.52	0.063
*adjusted for education level*, *migration* ^*c*^			
Education level			
*high*	Ref.		
*medium*	1.49	1.27–1.75	<0.001
*low*	2.75	2.18–3.46	<0.001
Migration			
*Non-immigrant*	Ref.		
*Resettlers*	1.50	1.00–2.25	0.048
*Turkish*	5.30	1.92–14.66	0.001
*Others*	1.57	0.96–2.57	0.073

*OR > 1 means greater odds for making an uninformed choice

^a^ n = 3,560

^b^ n = 3,599

^c^ n = 3,558

## Results

### Response and characteristics of study participants

5,847 (33.7%) women aged 50 years responded to the postal questionnaire. Women who already had breast cancer (n = 183), already participated in the national breast cancer screening program (n = 256), had not received an invitation for the screening program (n = 1,317), or had missing values on any of these variables were excluded. Thus the final dataset comprised 4,113 respondents. The numbers and proportion of missing values are displayed in Table 1.

### Intention to participate in mammography screening

Almost 74% of women in the sample intended to participate in the organized mammography screening program in the next three months (Table 1). 5.3% intended to receive opportunistic mammography screening, and 13.6% did not plan to participate. With increasing education level, more women intended not to participate (10.5% low, 13.4% medium, and 15.5% high education level). Fewer Turkish (60.6%) and other immigrant women (66.9%) intended to participate in the screening programme compared to non-immigrant women (74.6%) and resettlers (73.2%).

### Attitude

The mean attitude towards mammography in our study was positive overall (M = 4.24, SD = 2.72, possible score range -8–+8) as well as for all subgroups (Table 1). With increasing education level, the mean attitude score decreased while remaining in the positive spectrum. Non-immigrant women had a less positive mean attitude (M = 4.18, SD = 2.74) than immigrant women. Turkish women had the most positive mean attitude (M = 5.07, SD = 2.95).

### Knowledge

The mean knowledge score was 2.81 (SD = 1.44, possible range 0–7). All subgroup means, except for women of high education level, were below the scale midpoint indicating insufficient knowledge. Women with low education level on average answered fewer questions correctly (M = 2.23, SD = 1.32) than women of medium (M = 2.84, SD = 1.42) and of high education level (M = 3.14, SD = 1.42). Immigrant women answered fewer questions correctly than non-immigrant women. Turkish immigrant women (M = 1.73, SD = 1.12) had the lowest mean knowledge (Table 1).

### Informed choice

Table 2 shows the proportion of women categorized by the three dichotomous dimensions of informed choice. 25.6% of the women had sufficient knowledge combined with a positive attitude and an intention to perform mammography screening. 1.5% of the women had sufficient knowledge combined with a negative attitude and no intention to perform mammography. These groups were thus classified as making an informed choice. Most women (56.7%) had insufficient knowledge combined with a positive attitude and an intention to perform mammography screening.

Only 31.5% of the women had sufficient knowledge (Table 3). 86.4% of the women had an attitude consistent with their intention. There were significant differences in the proportion of sufficient knowledge by education level and migration status (Table 3). Only 27.1% of the women invited to the national mammography screening program could be categorized as having made an informed choice for or against participation (Table 3). The majority (72.9%) made an uninformed choice. With increasing education level, significantly larger proportions of women made informed choices (15.6% low, 26.5% medium, and 34.6% high education group). While 28.2% of non-immigrant women made an informed choice, 21.8% of Resettlers, 20.0% of other immigrants but only 5% of Turkish immigrant women made an informed choice (Table 3).

The results of the univariate logistic regression (Table 4) show that women of low education level had almost three times the odds of making an uninformed choice (OR 2.87, 95% CI 2.28–3.61) compared to women of high education level; women of medium education level had 1.47 times the odds (95% CI 1.25–1.73). Immigrant women had greater odds of making an uninformed choice compared to non-immigrant women: Turkish immigrant women had 7.44 (95% CI 2.72–20.40) times the odds (Table 4). Resettlers (OR 1.41, 95% CI 0.95–2.10) and other immigrant women (OR 1.57, 95% CI 0.98–2.52) also had greater odds of making an uninformed choice compared to non-immigrant women, though these differences are not statistically significant.

The results of the adjusted model showed that Turkish immigrant women still had greater odds of making an uninformed choice (OR 5.30, 95% CI 1.92–14.66) than non-immigrant women though the effect was smaller than in the unadjusted model. The odds of making an uninformed choice among resettlers (OR 1.50, 95% CI 1.00–2.25) were slightly higher than in the unadjusted model while the odds did not change for other immigrant women (OR 1.57, 95% CI 0.96–2.57).

## Discussion

This study estimates the proportion of women invited to the nation-wide mammography screening program in Germany for the first time who make an informed choice for or against participation. The most important findings are (I) the overall low proportion (27.1%) of informed choices; and (II) pronounced differences in the proportion of informed choice by education level and migration status. The overall low proportion of informed choices is explained by the low level of knowledge about mammography screening in our sample. Poor knowledge about mammography screening has been shown previously: women tend to overestimate the benefits and underestimate the risks of mammography screening [17, 18, 25]. Our study even shows a lower proportion of correct answers as found in a randomized, controlled trial evaluation the German information leaflet sent with the official invitation for the program [25]. This may be explained by a different study procedure. While women in the trial received the questionnaire and the information leaflet together, women in our study received it separately. We took this into account by defining a lower criterion for sufficient knowledge. Nonetheless, overall knowledge was very poor.

Research from the Netherlands, in contrast, showed much better knowledge levels among women eligible for mammography screening [19]. An explanation might be that more questions about organizational factors such as compulsory participation were included in their knowledge score and knowledge was measured by yes/no answers in that study. In our study, we only included one basic organizational item (‘Who is the target group of mammography screening?’) and had multiple answer options. All other questions related to a comparison of screening vs. no screening or methodological issues such as the concept of false-positive results. Since both type and number of knowledge-related questions as well as cut-off levels for “good” or “sufficient” knowledge differ between these studies, a comparison of the results is not possible.

As there are no clear guidelines as to what constitutes sufficient knowledge, we decided to use the mid-point as this has been applied successfully in previous research [19]. This, however, may be debatable. As different studies use different instruments and definitions, it is difficult to compare and synthesize the literature without access to the questions used. We added the German (S1 File) and Turkish (S2 File) questionnaires as additional materials to help readers make comparisons across studies. Regarding immigrant women our results show that resettlers and other immigrants only had slightly lower knowledge scores than non-immigrant women, in contrast to Turkish immigrant women whose knowledge was much lower. As Turkish immigrant women are known to have poor German language skills [26], we provided both German and Turkish questionnaires to women who were possibly Turkish. The invitation and information materials of the screening program, however, are provided with the official invitation in German only. Translated materials are available online and therefore unlikely to be accessed by Turkish migrant women [22]. In consequence, Turkish women with poor language skills might have been able to answer our questionnaire while not being able to understand the information material. This may explain the lower knowledge level, resulting in a lower proportion of informed choices especially among Turkish immigrant women. Most resettlers in contrast have good German language skills [27], which might explain the small difference to non-immigrant women. Among other immigrant women, we probably reached only those with adequate German language skills as the questionnaire was available only in German and Turkish.

Our results show a low participation rate in mammography screening among immigrant women which was also observed in other European countries [9–15]. Screening participation of Turkish immigrants in our sample was similar to that in previous research in Germany [16] but higher than the overall participation rate of 55% in the German screening program [1]. 3.5% of the women in our sample did not know whether they intend to participate or not and another 3.7% did not specify where they plan to perform mammography. This might be explained by asking about the intention to attend mammography in the next three months which we chose as time frame for participation according to the invitation letter appointment. Thereby, we possibly misclassified women who will go for mammography screening thereafter. Furthermore, intention does not necessarily reflect the actual behavior as there might be factors hindering intending women to participate. However, intention is suitable for inclusion in the concept of informed choice as actual behavior might be influenced by organizational barriers not affecting the decision *per se*.

The temporal restriction on intention might explain some inconsistencies between attitude and intention as shown in Table 2 which result in being categorized as uninformed choices. However, only women who had received an invitation were included in the analyses. This implies that they have a screening appointment in the next three to four weeks. Another reason for the inconsistencies might result from the way in which attitude was measured: the four semantic differentials of the Reasoned Action Approach [23] could not cover all favorable and unfavorable aspects of the mammography screening program.

Another limitation to our results is that the index of informed choice has not yet been validated. Our results support the validity of its components (attitude and knowledge), but due to a lack of other valid measures for informed choice in the German mammography screening program, we could not establish convergent validity. Future research is needed, especially regarding its predictive validity on decisional regret which was not included in our questionnaire.

A strength of our study is the focus on women invited to the nation-wide mammography screening program for the first time. Women received the study questionnaire close to the time of receiving the official invitation letter. Thus, the assessed knowledge level will reflect the actual knowledge at the time of decision taking, thereby minimizing recall bias.

The response rate of 33.7% is similar to that in other studies on mammography screening in Germany [18, 28, 29]. Women of higher education level are overrepresented in our study, compared to the distribution of education level among women in the German population [30]. The proportion of immigrant women in our study is fairly representative for resettlers and Turkish immigrant women; immigrants from other countries are underrepresented [30].

Future research should focus on the measurement of knowledge and attitude in mammography screening, as well as their determinants. There is also need for studies testing interventions to enhance knowledge levels and informed choice. As immigrant populations and women with low education level have been shown to have low levels of knowledge, they need special attention.

## Supporting Information

S1 File German questionnaire. (PDF)

S2 File Turkish questionnaire. (PDF)

## References

[1] Kooperationsgemeinschaft Mammographie. Evaluationsbericht 2008–2009 –Ergebnisse des Mammographie-Screening-Programms in Deutschland; 2012. Available: http://www.mammo-programm.de/download/Evaluationsbericht_Kooperationsgemeinschaft_Mammographie_08-09.pdf.

[2] Perry N, Broeders M, de Wolf C, Törnberg S, Holland R, von Karsa S. European guidelines for quality assurance in breast cancer screening and diagnosis. 4th ed Luxembourg: Office for Official Publications of the European Communities; 2006. 10.1093/annonc/mdm481 18024988

[3] Gøtzsche PC, Jørgensen KJ. Screening for breast cancer with mammography. Cochrane Database Syst Rev. 2013: 6 4;6:CD001877 10.1002/14651858.CD001877.pub5 23737396 PMC6464778

[4] Marteau TM, Dormandy E, Michie S. A measure of informed choice. Health Expect. 2001; 4: 99–108. 11359540 10.1046/j.1369-6513.2001.00140.x PMC5060053

[5] Helou A. Krebsfrüherkennung im Nationalen Krebsplan. Bundesgesundheitsbl. 2014; 57: 288–293. 10.1007/s00103-013-1902-3 24562702

[6] Lagerlund M, Maxwell AE, Bastani R, Thurfjell E, Ekbom A, Lambe M. Sociodemographic predictors of non-attendance at invitational mammography screening—a population-based register study (Sweden). Cancer Causes Control. 2002; 13: 73–82. 11899121 10.1023/a:1013978421073

[7] Puddu M, Demarest S, Tafforeau J. Does a national screening programme reduce socioeconomic inequalities in mammography use. Int J Public Health. 2009; 54: 61–68. 10.1007/s00038-009-8105-6 19247577

[8] Moser K, Patnick J, Beral V. Inequalities in reported use of breast and cervical screening in Great Britain: analysis of cross sectional survey data. BMJ. 2009; 338: b2025 10.1136/bmj.b2025 19531549 PMC2697310

[9] Fontana M, Bischoff A. Uptake of breast cancer screening measures among immigrant and Swiss women in Switzerland. Swiss Med Wkly. 2008; 138: 752–758. 19130329 10.4414/smw.2008.12328

[10] Bulliard JL, de LJP, Levi F. Profile of women not attending in the Swiss Mammography Screening Pilot. Breast. 2004; 13: 284–289. 10.1016/j.breast.2004.03.001 15325662

[11] Zackrisson S, Lindstrom M, Moghaddassi M, Andersson I, Janzon L. Social predictors of non-attendance in an urban mammographic screening programme. Scand J Public Health. 2007; 35: 548–554. 10.1080/14034940701291716 17852976

[12] Renshaw C, Jack RH, Dixon S, Moller H, Davies EA. Estimating attendance for breast cancer screening in ethnic groups in London. BMC Public Health. 2010; 10: 157 10.1186/1471-2458-10-157 20334699 PMC2850886

[13] Kristiansen M, Thorsted BL, Krasnik A, von Euler-Chelpin M. Participation in mammography screening among migrants and non-migrants in Denmark. Acta Oncol. 2012; 51: 28–36. 10.3109/0284186X.2011.626447 22035117

[14] Visser O, van Peppen AM, Ory FG, van Leuwen FE. Results of breast cancer screening in first generation migrants in Northwest. Eur J Cancer Prev. 2005; 14: 251–255. 15901994 10.1097/00008469-200506000-00009

[15] Vermeer B, Van den Muijsenbergh M. The attendance of migrant women at the national breast cancer screening in the Netherlands 1997–2008. Eur J Cancer Prev. 2010; 19: 195–198. 20150815 10.1097/CEJ.0b013e328337214c

[16] Berens E, Stahl L, Yilmaz-Aslan Y, Sauzet O, Spallek J, Razum O. Participation in breast cancer screening among women of Turkish origin in Germany—a register-based study. BMC Womens Health. 2014; 14: 24 10.1186/1472-6874-14-24 24507093 PMC3922307

[17] Gigerenzer G, Mata J, Frank R. Public knowledge of benefits of breast and prostate cancer screening in Europe. J. Natl. Cancer Inst. 2009; 101: 1216–1220. 10.1093/jnci/djp237 19671770 PMC2736294

[18] Diercks ML, Schmacke N. Mammografie-Screening und informierte Entscheidung–mehr Fragen als Antworten In: Böcken B, Braun B, Meierjürgen R, editors. Gesundheitsmonitor 2014. Bürgerorientierung im Gesundheitswesen. Gütersloh: Bertelsmann Stiftung; 2014 pp. 55–91.

[19] van Agt H, Fracheboud J, van der Steen A, de Koning H. Do women make an informed choice about participating in breast cancer screening? A survey among women invited for a first mammography screening examination. Patient Educ Couns. 2012; 89: 353–359. 10.1016/j.pec.2012.08.003 22963769

[20] Razum O, Zeeb H, Akgun S. How useful is a name-based algorithm in health research among Turkish migrants in Germany. Trop Med Int Health. 2001; 6: 654–661. 11555431 10.1046/j.1365-3156.2001.00760.x

[21] Berens E, Reder M, Kolip P, Spallek J. A cross-sectional study on informed choice in the mammography screening programme in Germany (InEMa): a study protocol. BMJ Open. 2014; 4: e006145 10.1136/bmjopen-2014-006145 25231495 PMC4166244

[22] Berens E, Yilmaz-Aslan Y, Spallek J, Razum O. Determinants of mammography screening participation among Turkish immigrant women in Germany–a qualitative study reflecting key informants' and women's perspectives. Eur J Cancer Care. 2015 10.1111/ecc.12334 26052964

[23] Fishbein M, Ajzen I. Predicting and changing behavior The reasoned action approach. New York: Psychology Press; 2010.

[24] Mathieu E, Barratt AL, McGeechan K, Davey HM, Howard K, Houssami N. Helping women make choices about mammography screening: An online randomized trial of a decision aid for 40-year-old women. Patient Education and Counseling. 2010; 81: 63–72. 10.1016/j.pec.2010.01.001 20149953

[25] Gummersbach E, in der Schmitten J, Mortsiefer A, Abholz HH, Wegscheider K, Pentzek M. Willingness to participate in mammography screening. A randomized controlled questionnaire study of responses to two patient information leaflets with different factual content. Dtsch Arztebl Int. 2015; 112: 61–8. 10.3238/arztebl.2015.0061 25686383 PMC4335580

[26] Haug S. Sprachliche Integration von Migranten in Deutschland. Nürnberg: Bundesamt für Migration und Flüchtlinge; 2008.

[27] Worbs S, Bund E, Kohls M, Babka von Gostomski C. (Spät)-Aussiedler in Deutschland: Eine Analyse aktueller Daten und Forschungsergebnisse Forschungsbericht 20. Nürnberg: Bundesamt für Migration und Flüchtlinge; 2013.

[28] Klug SJ, Hetzer M, Blettner M. Screening for breast and cervical cancer in a large German city: participation, motivation and knowledge of risk factors. Eur J Public Health. 2005; 15: 70–77. 10.1093/eurpub/cki118 15788807

[29] Nass-Griegoleit I, Schultz-Zehden B, Klusendick M, Diener J, Schulte H. Studie belegt hohe Akzeptanz des Mammographie-Screenings bei Frauen. Frauenarzt. 2009; 50: 494–501.

[30] Statistisches Bundesamt. Bevölkerung mit Migrationshintergrund—Ergebnisse des Mikrozensus 2011—Fachserie 1 Reihe 2.2; 2012. Available: https://www.destatis.de/DE/Publikationen/Thematisch/Bevoelkerung/MigrationIntegration/Migrationshintergrund2010220117004.pdf?__blob=publicationFile.

