# Supplementary material for: Informed Choice in the German Mammography Screening Program by Education and Migrant Status: Survey among First-Time Invitees
Source: PLoS One. 2015 Nov 3;10(11):e0142316. doi: 10.1371/journal.pone.0142316 (PMC4631499; doi:10.1371/journal.pone.0142316)
Supplement: S1 File — (PDF) [file pone.0142316.s001.pdf]

A. Entscheidungsfindung

1. Ich habe vor das Mammographie-Screening in den nächsten 3 Monaten...

durchführen zu lassen . . . . ☐
nicht durchführen zu lassen . . . . ☐ → *Bitte weiter mit* ***Frage*** 3

2. Wo werden Sie das Mammographie-Screening durchführen lassen?

- ☐ Bei einer niedergelassenen Ärztin/einem niedergelassenen Arzt (Gynäkologie, Radiologie, Hausarztpraxis)
- ☐ Im Rahmen einer Einladung zum Mammographie-Screening-Programm

3. Bei meiner Entscheidung bin ich mir...

Sicher				Unsicher
☐	☐	☐	☐	☐
1	2	3	4	5

4. Wie schätzen Sie Ihr Wissen über Mammographie-Screening ein?

Sehr gut				Sehr schlecht
☐	☐	☐	☐	☐
1	2	3	4	5

B. Erreichbarkeit der Screening-Einheit

5. Wie weit ist die Screening-Einheit von Ihrem Wohnort entfernt?

- ☐ Weniger als 9 Minuten
- ☐ 10 bis 19 Minuten
- ☐ 20 bis 39 Minuten
- ☐ 40 bis 60 Minuten
- ☐ Weiter als 60 Minuten
- ☐ Weiß nicht, wo die Screening-Einheit ist

©Bielefeld University

C. Persönliche Einstellung

6. Am Mammographie-Screening-Programm teilzunehmen ist...
Bitte kreuzen Sie jeweils an, wie Sie die Teilnahme bewerten.

Wichtig	☐	☐	☐	☐	☐	Unwichtig
	1	2	3	4	5	
Eine gute Sache	☐	☐	☐	☐	☐	Eine schlechte Sache
	1	2	3	4	5	
Angenehm	☐	☐	☐	☐	☐	Unangenehm
	1	2	3	4	5	
Vorteilhaft	☐	☐	☐	☐	☐	Nachteilig
	1	2	3	4	5	

D. Gesundheitszustand und Gesundheitsverhalten

7. Wie beurteilen Sie Ihren Gesundheitszustand im Allgemeinen?

Sehr gut	☐	☐	☐	☐	☐	Sehr schlecht
	1	2	3	4	5	

8. Nutzen Sie folgende Angebote zur Früherkennung von Krankheiten?

	Ja, regelmäßig	Ja, gelegentlich	Selten	Nie
Gynäkologische Krebsfrüherkennung („Vorsorgeuntersuchung“ beim Frauenarzt)	☐	☐	☐	☐
Hautkrebsfrüherkennung	☐	☐	☐	☐
Gesundheits-Check-Up	☐	☐	☐	☐

©Bielefeld University

9. Wurde bei Ihnen schon mal eine Mammographie gemacht?

Ja . . . . ☐
Nein . . . . ☐ → *Bitte weiter mit **Frage** 11*

10. Was war der Grund für die letzte Mammographie?

- ☐ Vorsorge/Früherkennung
- ☐ Probleme/Beschwerden
- ☐ Weiß nicht

11. Führen Sie systematische Selbstuntersuchungen (Tastuntersuchung) Ihrer Brust durch?

- ☐ Ja, regelmäßig
- ☐ Ja, gelegentlich
- ☐ Nein

12. Rauchen Sie zurzeit?

- ☐ Ja, regelmäßig
- ☐ Ja, gelegentlich
- ☐ Nein, nicht mehr
- ☐ Nein, ich habe noch nie geraucht

©Bielefeld University

E. Umstände, die Ihre Entscheidung beeinflussen

13. Stimmen Sie folgenden Aussagen zu?

	Stimme zu				Stimme nicht zu
Ich finde es unangenehm, wenn während der Untersuchung mein Körper berührt wird.	☐	☐	☐	☐	☐
Ich habe Angst vor Schmerzen während des Mammographie-Screenings.	☐	☐	☐	☐	☐
Ich habe widersprüchliche Empfehlungen zum Mammographie-Screening-Programm bekommen.	☐	☐	☐	☐	☐
Ich sehe den Krankheitsverlauf als vorherbestimmt an.	☐	☐	☐	☐	☐
Ich will lieber nicht wissen, wenn etwas nicht stimmt.	☐	☐	☐	☐	☐
Ich bin unsicher, was mich erwartet.	☐	☐	☐	☐	☐
Die Strahlendosis beim Mammographie-Screening ist schädlich.	☐	☐	☐	☐	☐
Ich fühle mich durch die Einladung verpflichtet.	☐	☐	☐	☐	☐
Ich habe Vertrauen in das Mammographie-Screening-Programm.	☐	☐	☐	☐	☐
Sonstiges, und zwar ________________	☐	☐	☐	☐	☐

©Bielefeld University

14. Stimmen Sie folgenden Aussagen zu?

	Stimme zu				Stimme nicht zu
Ich habe andere Probleme, die wichtiger sind als eine Mammographie.	☐	☐	☐	☐	☐
Ich habe keine Zeit für den Termin.	☐	☐	☐	☐	☐
Ich bin im Urlaub/außer Landes.	☐	☐	☐	☐	☐
Ich habe Sprachprobleme.	☐	☐	☐	☐	☐
Die finanziellen Kosten sind mir zu hoch.	☐	☐	☐	☐	☐
Ich habe Probleme, zur Screening-Einheit zu kommen.	☐	☐	☐	☐	☐
Sonstiges, und zwar ________________	☐	☐	☐	☐	☐

F. Rolle anderer Menschen

15. Haben Ihnen folgende Personen einen Rat für oder gegen die Teilnahme am Mammographie-Screening-Programm gegeben?

	Zuge-raten		Teils/ teils		Abge-raten	*Keinen Rat*
Meine Gynäkologin/mein Gynäkologe	☐	☐	☐	☐	☐	☐
Meine Hausärztin/mein Hausarzt	☐	☐	☐	☐	☐	☐
Mein Partner/Ehemann/meine Partnerin	☐	☐	☐	☐	☐	☐
Meine Verwandten	☐	☐	☐	☐	☐	☐
Meine Freundinnen/Freunde/Bekannten	☐	☐	☐	☐	☐	☐

©Bielefeld University

G. Wissen

16. Wann nimmt man an einem Mammographie-Screening teil?

- ☐ Wenn man gesund ist
- ☐ Wenn man eine Veränderung oder einen Knoten in der Brust bemerkt
- ☐ In beiden Fällen
- ☐ Weiß nicht

17. Bitte stellen Sie sich Folgendes vor: 200 Frauen nehmen 20 Jahre lang am Mammographie-Screening-Programm teil. Bei wie vielen Frauen wird im Rahmen des Screenings eine Auffälligkeit entdeckt, die abgeklärt werden muss (positiver Befund)?

- ☐ 1-20 von 200
- ☐ 21-50 von 200
- ☐ 51-100 von 200
- ☐ 101-200 von 200
- ☐ Weiß nicht

18. Bedeutet ein positiver Befund beim Mammographie-Screening, dass diese Frau Brustkrebs hat?

- ☐ Ja
- ☐ Nein
- ☐ Weiß nicht

19. Wird beim Mammographie-Screening jeder Brustkrebs entdeckt?

- ☐ Ja
- ☐ Nein
- ☐ Weiß nicht

20. Wer bekommt mit größerer Wahrscheinlichkeit die <u>Diagnose</u> Brustkrebs?

- ☐ Frauen, die am Mammographie-Screening teilnehmen
- ☐ Frauen, die <u>nicht</u> am Mammographie-Screening teilnehmen
- ☐ Beide gleich
- ☐ Weiß nicht

21. Wer stirbt mit größerer Wahrscheinlichkeit an Brustkrebs?

- ☐ Frauen, die am Mammographie-Screening teilnehmen
- ☐ Frauen, die <u>nicht</u> am Mammographie-Screening teilnehmen
- ☐ Beide gleich
- ☐ Weiß nicht

©Bielefeld University

22. Gibt es Frauen, die wegen Brustkrebs behandelt werden, obwohl der Brustkrebs in ihrem Leben nie Probleme verursacht hätte?

- ☐ Ja
- ☐ Nein
- ☐ Weiß nicht

H. Fragen im Zusammenhang mit Brustkrebs

23. Für wie wahrscheinlich halten Sie es, dass Sie eines Tages Brustkrebs bekommen?

Sehr unwahrscheinlich				Sehr wahrscheinlich
☐	☐	☐	☐	☐
1	2	3	4	5

24. Ist bei Ihnen jemals eine Brustkrebserkrankung festgestellt worden?

- ☐ Ja
- ☐ Nein

25. Hatte oder hat Ihre Mutter oder eine Schwester eine Brustkrebserkrankung?

- ☐ Ja
- ☐ Nein

I. Persönlicher Hintergrund

26. Haben Sie einen festen Partner/eine feste Partnerin?

- ☐ Ja
- ☐ Nein

27. Welchen höchsten allgemeinbildenden Schulabschluss haben Sie?

- ☐ Haupt- oder Volksschulabschluss
- ☐ Realschulabschluss/Mittlere Reife/Fachschulreife
- ☐ POS (Polytechn. Oberschule) bzw. 10. Klasse
- ☐ Fachhochschulreife/Abschluss einer Fachoberschule
- ☐ Abitur, allgemeine oder fachgebundene Hochschulreife
- ☐ Anderen Schulabschluss (z.B. im Ausland erworben) mit ☐☐ Schuljahren
- ☐ Schule beendet ohne Schulabschluss

©Bielefeld University

28. **In welchem Land sind Sie geboren?**
Bitte verwenden Sie die heutige Staatsbezeichnung, auch wenn das Gebiet früher einem anderen Staat zugeordnet war.

- ◻ Deutschland (auf dem Gebiet der ehemaligen DDR)
- ◻ Deutschland (auf dem ehemaligen Gebiet der BRD)
- ◻ Türkei
- ◻ Bosnien-Herzegowina
- ◻ Griechenland
- ◻ Italien
- ◻ Kroatien
- ◻ Mazedonien
- ◻ Polen
- ◻ Russland
- ◻ Slowenien
- ◻ Spanien
- ◻ In einem anderen Land, und zwar______________________________

29. **Seit wann leben Sie auf dem heutigen Gebiet der Bundesrepublik Deutschland (einschließlich der ehemaligen DDR)?**

- ◻ Seit meiner Geburt
- ◻ Seit ☐☐ Jahren

30. **Besitzen Sie die deutsche Staatsangehörigkeit?**

- ◻ Ja, durch Geburt
- ◻ Ja, als (Spät-)Aussiedlerin ohne Einbürgerung
- ◻ Ja, als (Spät-)Aussiedlerin mit Einbürgerung
- ◻ Ja, durch Einbürgerung
- ◻ Nein

©Bielefeld University

31. Welche Sprache wird bei Ihnen zu Hause hauptsächlich gesprochen?
Mehrfachantworten sind möglich.

- ☐ Deutsch
- ☐ Türkisch
- ☐ Bosnisch
- ☐ Griechisch
- ☐ Italienisch
- ☐ Kroatisch
- ☐ Mazedonisch
- ☐ Polnisch
- ☐ Russisch
- ☐ Slowenisch
- ☐ Spanisch
- ☐ Arabisch
- ☐ Andere Sprache und zwar ________________________________

32. Wie viele Einwohner hat Ihr Wohnort?

- ☐ Unter 1.000 Einwohner
- ☐ 1.000 bis unter 5.000 Einwohner
- ☐ 5.000 bis unter 10.000 Einwohner
- ☐ 10.000 bis unter 30.000 Einwohner
- ☐ 30.000 bis unter 100.000 Einwohner
- ☐ 100.000 Einwohner oder mehr

33. Welche Krankenversicherung bzw. -versorgung haben Sie?
Mehrfachantworten sind möglich.

- ☐ Gesetzliche Krankenversicherung (GKV)
- ☐ Beihilfe
- ☐ Private Krankenversicherung als Vollversicherung
- ☐ Private Krankenversicherung als Zusatzversicherung
- ☐ Ausländische Krankenkasse
- ☐ Sonstiger Anspruch auf Krankenversorgung (z.B. freie Heilfürsorge, Sozialhilfeempfänger)
- ☐ Keine Krankenversicherung, Selbstzahler
- ☐ Falls Sie nicht wissen, wie Ihre Krankenversicherung einzuordnen ist, schreiben Sie bitte den genauen Namen der Krankenkasse auf:________________________________

©Bielefeld University

34. Nehmen Sie an einem Bonus- oder Punkteprogramm Ihrer Krankenkasse teil?
Diese Programme dienen zur Förderung von Vorsorge- und Früherkennungsmaßnahmen. Bei manchen Krankenkassen ist die Teilnahme automatisch, bei anderen müssen Sie sich zunächst registrieren. Manche Krankenkassen bieten dies nicht an.

Ja . . . . ☐
Nein . . . . ☐ → *Bitte weiter mit* ***Frage*** *37*
Weiß nicht . . . . ☐ → *Bitte weiter mit* ***Frage*** *37*

35. Beinhaltet dieses Bonus- oder Punkteprogramm auch die Teilnahme am Mammographie-Screening-Programm?

Ja . . . . ☐
Nein . . . . ☐ → *Bitte weiter mit* ***Frage*** *37*
Weiß nicht . . . . ☐ → *Bitte weiter mit* ***Frage*** *37*

36. Was für einen Bonus/eine Prämie würden Sie für eine Teilnahme am Mammographie-Screening-Programm bekommen?
Wählen Sie auch dann eine Möglichkeit aus, wenn Sie zunächst noch weitere Punkte sammeln müssen.

- ☐ Geldprämie oder Erstattung der Versicherungsbeiträge
- ☐ Geldwerte Prämie wie z.B. einen Gutschein oder eine Zusatzversicherung
- ☐ Erlebnisprämie wie z.B. einen Yogakurs
- ☐ Sachprämie wie z.B. einen Volleyball oder ein Buch
- ☐ Weiß nicht

37. Wie schätzen Sie Ihre Deutschkenntnisse in folgenden Bereichen ein?

	Keine Kennt-nisse				Sehr gute Kennt-nisse
Hören und Verstehen	☐	☐	☐	☐	☐
Sprechen	☐	☐	☐	☐	☐
Lesen	☐	☐	☐	☐	☐
Schreiben	☐	☐	☐	☐	☐

 ©Bielefeld University

J. Fragen zu Fragebogen und Einladung

38. Haben Sie eine Einladung zum Mammographie-Screening-Programm erhalten?

Ja . . . . ☐
Nein . . . . ☐ → *Bitte weiter mit **Frage** 40*

39. Wann haben Sie diesen Fragebogen erhalten?

- ☐ Vor der Einladung zum Mammographie-Screening-Programm und zwar ☐☐ Tag(e) davor
- ☐ Am gleichen Tag
- ☐ Nach der Einladung zum Mammographie-Screening-Programm und zwar ☐☐ Tag(e) danach

40. Inwieweit hat die Befragung Ihre Entscheidung für oder gegen das Mammographie-Screening beeinflusst?

Gar nicht				Sehr stark
☐	☐	☐	☐	☐
1	2	3	4	5

41. Gibt es noch etwas, das Sie uns mitteilen möchten?

____

____

____

Vielen herzlichen Dank!

Sie helfen uns mit Ihren Antworten sehr. Wir freuen uns, dass Sie sich die Zeit für unseren Fragebogen genommen haben.

Bitte schicken Sie nun den Fragebogen zusammen mit Ihrer Einverständniserklärung an uns zurück.

©Bielefeld University
