# Supplementary material for: Informed Choice in the German Mammography Screening Program by Education and Migrant Status: Survey among First-Time Invitees
Source: PLoS One. 2015 Nov 3;10(11):e0142316. doi: 10.1371/journal.pone.0142316 (PMC4631499; doi:10.1371/journal.pone.0142316)
Supplement: S2 File — (PDF) [file pone.0142316.s002.pdf]

A. Karar verme aşaması

1. Mamografi taraması programına önümüzdeki üç ay içinde...

Katılacağım . . . . ☐
Katılmayacağım . . . . ☐ → *Soru 3ile devam ediniz lütfen.*

2. Mamografi taramasını nerde gerçekleştirmeyi düşünüyorsunuz?

- ☐ Muayenehanesi olan bir doktorda (jinekoloji, radyoloji, ev doktoru)
- ☐ Mamografi taramasına gelen davetiye mektubunda belirtilen yerde

3. Kararımı verirken...

Eminim				Emin değilim
☐	☐	☐	☐	☐
1	2	3	4	5

4. Mamografi taraması hakkında sahip olduğunuz bilgiyi nasıl değerlendiriyorsunuz?

Çok iyi				Çok kötü
☐	☐	☐	☐	☐
1	2	3	4	5

B. Mamografi taramasına ulaşım imkanı

5. Mamografi taramasının bulunduğu yere eviniz ne kadar uzak?

- ☐ 9 dakikadan daha az
- ☐ 10 ile 19 dakika arası
- ☐ 20 ile 39 dakika arası
- ☐ 40 ile 60 dakika arası
- ☐ 60 dakikadan daha fazla
- ☐ Taramanın nerde gerçekleşeceğini bilmiyorum

©Bielefeld University

C. Kişisel görüşleriniz

6. Mamografi taraması porgramına katılmak...
Lütfen katılımı nasıl değerlendirdiğinizi belirten cevabı seçiniz.

Önemli	☐	☐	☐	☐	☐	Önemsiz
	1	2	3	4	5	
İyi birşey	☐	☐	☐	☐	☐	Kötü birşey
	1	2	3	4	5	
Hoş	☐	☐	☐	☐	☐	Hoş değil
	1	2	3	4	5	
Faydalı	☐	☐	☐	☐	☐	Faydasız
	1	2	3	4	5	

D. Sağlık durumunuz ve sağlık tutumunuz

7. Genel olarak sağlık durumunuzu nasıl nitelendiriyorusunuz?

Çok iyi	☐	☐	☐	☐	☐	Çok kötü
	1	2	3	4	5	

8. Hastalığa yönelik erken teşhis için sunulan teklifleri değerlendiriyor musunuz?

	Evet düzenli bir şekilde	Evet, ara sıra	Nadiren	Hiç
Jinekolojik hastalıklar için erken teşhis muayenesi (Kadın doktorunda ihtiyati muayene)	☐	☐	☐	☐
Cilt kanseri için erken teşhis muayenesi	☐	☐	☐	☐
Genel sağlık kontrolü için Check-Up	☐	☐	☐	☐

©Bielefeld University

9. Bugüne kadar hiç bir mamografi taramasına katıldınız mı?

Evet . . . . ☐
Hayır . . . . ☐ → ***Soru*** 11 *ile devam ediniz lüften*

10. En son katılmış olduğunuz mamografinin sebebi neydi?

- ☐ Önlem/Erken teşhis
- ☐ Sorun/Şikayet
- ☐ Bilmiyorum

11. Kendi kendinize meme muayenesi yapıyor musunuz?

- ☐ Evet, düzenli bir şekilde
- ☐ Evet, ara sıra
- ☐ Hayır

12. Sigara kullanıyor musunuz?

- ☐ Evet, devamlı kullanıyorum
- ☐ Evet, ara sıra
- ☐ Hayır, artık kullamıyorum
- ☐ Hayır, hiç sigara kullanmadım

©Bielefeld University

E. Kararınızı etkileyen unsurlar

13. Aşağıdaki ifadelere katılıyor musunuz?

	Katılıyorum				Katılmıyorum
Muayene esnasında vücuduma dokunulmasından hoşlanmıyorum.	☐	☐	☐	☐	☐
Mamografi taraması esnasında oluşacak acıdan korkuyorum	☐	☐	☐	☐	☐
Mamografi taraması programı hakkında birbirine çok zıt düşüncelerle karşılaştım.	☐	☐	☐	☐	☐
Hastalığın gidişatı zaten önceden belli.	☐	☐	☐	☐	☐
Kötü bir sonuç olduğunda öğrenmemek tercihim.	☐	☐	☐	☐	☐
Mamografi taramasında neyle karşılaşacağımı bilmiyorum.	☐	☐	☐	☐	☐
Mamografi taramasında maruz kaldığım ışığın dozu zararlı.	☐	☐	☐	☐	☐
Davet edildiğim için kendimi katılmak zorundaymışım gibi hissediyorum.	☐	☐	☐	☐	☐
Mamografi taraması programına güveniyorum.	☐	☐	☐	☐	☐
Başka şeyler, mesela ________________	☐	☐	☐	☐	☐

©Bielefeld University

14. Aşağıdaki ifadelere katılıyor musunuz?

	Katılıyorum				Katılmıyorum
Mamografiden daha önemli sorunlarım var.	☐	☐	☐	☐	☐
Görüşmeye gitmek için vaktim yok.	☐	☐	☐	☐	☐
Görüşme tarihinde yurtdışında tatilde olacağım.	☐	☐	☐	☐	☐
Yabancı dil sorunum var.	☐	☐	☐	☐	☐
Maddi harcamalar benim için çok yüksek.	☐	☐	☐	☐	☐
Mamografi taramasının gerçekleşeceği yere ulaşmak benim için sorun.	☐	☐	☐	☐	☐
Başka şeyler, mesela ______________	☐	☐	☐	☐	☐

F. Başka kişilerin etkisi

15. Ağağıdaki kişilerden herhangi biri size mamografi taraması programına yönelik olumlu veya olumsuz tavsiyede bulundu mu?

	Tavsiye etti		Kısmen		Tavsiye etmedi	*Hiç tavsiyede bulunmadı*
Jinekoloğum	☐	☐	☐	☐	☐	☐
Ev doktorum	☐	☐	☐	☐	☐	☐
Partnerim/eşim	☐	☐	☐	☐	☐	☐
Akrabalarım	☐	☐	☐	☐	☐	☐
Arkadaşlarım/tanıdıklarım	☐	☐	☐	☐	☐	☐

©Bielefeld University

G. Mamografi taramasına yönelik bilgileriniz

16. Mamografi taramasına ne zaman katılmalı?

- ☐ Sağlıklı olunca
- ☐ Göğüsde bir sorun veya kitle farkedildiğinde
- ☐ Her iki durumda
- ☐ Bilmiyorum

17. Lütfen şu durumu düşününüz: 200 kadın 20 yıl boyunca mamografi taraması programına katılmaktadır. Sizce tarama çercevesinde kaç kadının göğsünde açıklanması gereken farklılığa (pozitif bulgu) rastlanmaktadır?

- ☐ 200 kişiden 1-20 kişide
- ☐ 200 kişiden 21-50 kişide
- ☐ 200 kişiden 51-100 kişide
- ☐ 200 kişiden 101-200 kişide
- ☐ Bilmiyorum

18. Mamografi taramasında ortaya çıkan pozitif bulgu kadınların göğüs kanserine yakalandığını doğrular mı?

- ☐ Evet
- ☐ Hayır
- ☐ Bilmiyorum

19. Mamografi taramasında her göğüs kanseri teşhis ediliyor mu?

- ☐ Evet
- ☐ Hayır
- ☐ Bilmiyorum

20. Kimlerin göğüs kanseri teşhisi ile karşılaşma olasılığı daha yüksektir?

- ☐ Mamografi taramasına katılan kadınların
- ☐ Mamografi taramasına katılmayan kadınların
- ☐ İkisininde de aynı
- ☐ Bilmiyorum

21. Kimlerin göğüs kanserine yakalanıp ölme ihtimali daha yüksektir?

- ☐ Mamografi taramasına katılan kadınların
- ☐ Mamografi taramasına katılmayan kadınların
- ☐ İkisininde de aynı
- ☐ Bilmiyorum

©Bielefeld University

22. Göğüs kanserine yakalanan fakat kanserin günlük hayatında hiç sorun yaratmadığı halde göğüs kanseri tedavisi gören kadınlar var mıdır?

- ☐ Evet
- ☐ Hayır
- ☐ Bilmiyorum

H. Göğüs kanseri ile ilgili sorular

23. Bir gün göğüs kanserine yakalanma riskiniz sizce ne kadar muhtemel?

Hiç muhtemel değil				Çok muhtemel
☐	☐	☐	☐	☐
1	2	3	4	5

24. Sizde hiç göğüs kanseri teşhis edildi mi?

- ☐ Evet
- ☐ Hayır

25. Anneniz veya kız kardeşinizde göğüs kanseri teşhis edildi mi?

- ☐ Evet
- ☐ Hayır

I. Kişisel bilgileriniz

26. Sabit bir partneriniz var mı?

- ☐ Evet
- ☐ Hayır

27. Eğitim durumunuz nedir?

- ☐ Ortaokul mezunu (Haupt- oder Volksschulabschluss)
- ☐ Ortaokul mezunu (Realschulabschluss/ Mittlere Reife/ Fachschulreife)
- ☐ Politeknik okul mezunu (POS (Polytechn. Oberschule) ya da 10.sınıf sonrası mezuniyet)
- ☐ Meslek lisesi mezunu (Fachhochschulreife/ Abschluss einer Fachoberschule)
- ☐ Genel lise mezunu (Abitur, allgemeine oder fachgebundene Hochschulreife)
- ☐ Herhangi başka bir mezuniyet derecesi (örneğin yurtdışında alınan) ☐☐ yaşındayken
- ☐ Mezun olmadan bitirilen bir okul eğitimi

©Bielefeld University

28. Hangi ülkede doğdunuz?
Eğer eskiden doğdunuz bölge başka bir devlete bağlı olmuş olsa da, lütfen bugünün devlet adlandırmasını kullanınız.

- ◻ Almanya (geçmis DDR bölgesinde)
- ◻ Almanya (geçmis BRD bölgesinde)
- ◻ Türkiye
- ◻ Bosna-Hersek
- ◻ Yunanistan
- ◻ Italya
- ◻ Hırvatistan
- ◻ Makedonya
- ◻ Polonya
- ◻ Rusya
- ◻ Slowenya
- ◻ Ispanya
- ◻ Başka bir ülkede, yani ______________________________

29. Ne zamandan beri Almanya' da yaşıyorsunuz?

- ◻ Doğumumdan itibaren
- ◻ ☐☐ senesinden itibaren

30. Alman vatandaşı mısınız?

- ◻ Evet, burda doğmuş olmamdan dolayı
- ◻ Evet, Alman kökenli göçmen olarak (vatandaşlığa kabul edilmeden)
- ◻ Evet, Alman kökenli göçmeni olarak (vatandaşlığa kabul edilip)
- ◻ Evet, alman vatandaşlığına geçerek
- ◻ Hayır

©Bielefeld University

31. Evde hangi dili konuşuyorsunuz?
Birden fazla cevap verebilirsiniz.

- ☐ Almanca
- ☐ Türkçe
- ☐ Boşnakca
- ☐ Yunanca
- ☐ Italyanca
- ☐ Hırvatça
- ☐ Makedonca
- ☐ Lehçe
- ☐ Rusça
- ☐ Slovence
- ☐ Ispanyolca
- ☐ Arapça
- ☐ Başka dil, yani ______________________________

32. Yaşadığınız yerin nüfusu kaçtır?

- ☐ 1.000 kişiden az
- ☐ 1.000 ile 5.000 kişi arası
- ☐ 5.000 ile 10.000 kişi arası
- ☐ 10.000 ile 30.000 kişi arası
- ☐ 30.000 ile 100.000 kişi arası
- ☐ 100.000 kişiden daha fazla

33. Hangi sağlık sigortasına bağlısınız?
Birden fazla cevap verebilirsiniz.

- ☐ Yasal sağlık sigortası (Gesetzliche Krankenversicherung - GKV) örneğin: AOK, IKK, TKK
- ☐ Ek yardım (Beihilfe)
- ☐ Tam sigorta olarak özel sağlık sigortası (Private Krankenversicherung als Vollversicherung)
- ☐ Ek sigorta olarak özel sağlık sigortası (Private Krankenversicherung als Zusatzversicherung)
- ☐ Yurtdışında sigortalı (Ausländische Krankenkasse)
- ☐ Herhangi bir başka sağlık sigortası hakkına sahip (örneğin: freie Heilfürsorge, Sozialhilfeempfänger)
- ☐ Sağlık sigortam yok, kendim ödüyorum
- ☐ Eğer sağlık sigortanız hakkında bir bilginiz yoksa, bu durumda hangi sağlık sigortası şirketine bağlı olduğunuzu yazmanız yeterli:______________________________

©Bielefeld University

34. Sağlık sigortanızın sunduğu bonus veya puan programını değerlendiriyor musunuz?
Bu programlar önlem ve erken teşhisi desteklemek için vardır. Bazı sağlık sigortalarında katılım otomatik gerçekleşiyor, bazılarında ise önce kayıt olmanız gerekir. Bazı sağlık sigortaları ise böyle programlar sunmuyor.

Evet . . . . ☐
Hayır. . . . .☐ → *Soru* 37*ile devam ediniz lütfen.*
Bilmiyorum. . . . .☐ → *Soru* 37*ile devam ediniz lütfen.*

35. Bonus ve puan programında mamografi taraması programına katılım dahil midir?

Evet . . . . ☐
Hayır. . . . .☐ → *Soru* 37*ile devam ediniz lütfen.*
Bilmiyorum. . . . .☐ → *Soru* 37*ile devam ediniz lütfen.*

36. Mamografi taraması programına katıldığınızda nasıl bir ödül isterdiniz?
Eğer henüz puan toplamanız gerekiyorsa ödüllendirilmek için, bu durumda da aşağıdaki cevaplardan birini seçiniz.

- ☐ Para ödülü veya sigorta üçretinin bir kısmını geri almak
- ☐ Parasal değeri olan ödüller, örneğin: hediye çeki veya ek sigorta
- ☐ Yaşayabileceğiniz bir ödül, örneğin: yoga kursu
- ☐ Eşya ödülü, örneğin: voleybol topu veya kitap
- ☐ Bilmiyorum

37. Aşağıda yazılı olan alanlarda almanca bilgilerinizi nasıl değerlendiriyorsunuz?

	Hiç bilmiyorum				Çok iyi biliyorum
Duymak ve anlamak	☐	☐	☐	☐	☐
Konuşmak	☐	☐	☐	☐	☐
Okumak	☐	☐	☐	☐	☐
Yazmak	☐	☐	☐	☐	☐

©Bielefeld University

J. Ankete ve davetiyeye yönelik sorular

38. Mamografi taraması programına davet edildiniz mi?

Evet . . . . ☐
Hayır. . . . .☐ → *Soru* 40 *ile devam ediniz lütfen.*

39. Bu anket elinize ne zaman geçti?

☐ Mamografi taraması programına davet edilmeden ☐☐ gün önce

☐ Mamografi taraması programına davet edildiğim gün

☐ Mamografi taraması programına davet edildikten sonra, yani ☐☐ gün sonra

40. Mamografi taraması hakkında olan düşüncelerinizi ne kadar etkiledi bu anket?

Hiç				Çok
☐	☐	☐	☐	☐
1	2	3	4	5

41. Bizimle paylaşmak istediğiniz başka birşey var mı?

____

____

____

Çok teşekkür ederiz!

Cevaplarınız bize çok yardımcı olacaktır. Anketimize zaman ayırdığınız için çok teşekkür ediyoruz.

Lütfen doldurduğunuz anketi imzalamış olduğunuz katılım onayı ile birlikte bize geri gönderiniz.

©Bielefeld University
